# Effects of apple juice on risk factors of lipid profile, inflammation and coagulation, endothelial markers and atherosclerotic lesions in high cholesterolemic rabbits

**DOI:** 10.1186/1476-511X-8-39

**Published:** 2009-10-05

**Authors:** Mahbubeh Setorki, Sedighe Asgary, Akram Eidi, Ali Haeri rohani, Nafiseh Esmaeil

**Affiliations:** 1 Department of Biology, Science and Research Branch, Islamic Azad University, Tehran, Iran; 2 Isfahan Cardiovascular Research Center, Applied Physiology Research Center, Isfahan University of Medical Sciences, Isfahan, Iran; 3 Department of immunology, Isfahan University of Medical Sciences, Isfahan, Iran

## Abstract

**Background:**

Atherosclerosis which results from gradual deposition of lipids in medium and large arteries is a leading cause of mortality worldwide. The objective of this study was to determine the effect of apple juice on some risk factors of atherosclerosis and on the development of atherosclerosis in rabbits fed a high-cholesterol diet.

**Methods:**

Thirty two male rabbits were randomly divided into four groups: normal diet, high cholesterol diet (%1 cholesterol), 1% cholesterol supplemented with 5 ml apple juice (low dose) and 1% cholesterol supplemented with 10 ml apple juice (high dose) for 2 month. The C-reactive protein (CRP), nitrite, nitrate, fibrinogen, total cholesterol(TC) and factor VII were measured before the experiment and by the end of period. At the end of study, fatty streak formation in right and left coronary arteries were determined using Chekanov method in all groups.

**Results:**

Both doses of apple juice significantly were decreased TC, TG, CRP, fibrinogen, factor VII levels, atherosclerotic lesion in right and left coronary arteries and increased nitrite and nitrate compared to cholesterolemic diet. Also using 10 ml apple juice caused significant reduce in LDL-C and increase HDL-C, but 5 ml apple juice did not change these factors. Significant differences were observed between 5 and 10 ml apple juice groups by LDL-C. No significant difference was found between 5 and 10 ml apple juice groups with regard to CRP, nitrite, nitrate, fibrinogen, factor VII, TG, HDL-C and TC concentrations.

**Conclusion:**

Apple juice can effectively prevent the progress of atherosclerosis. This is likely due to antioxidant and anti-inflammatory effect of apple juice.

## Introduction

Atherosclerosis is the leading cause of mortality in developed countries. This complex disease can be described as an excessive inflammatory, fibro fatty, proliferate response to damage of the artery wall. Many believe that it can be induced from simple dysfunction of endothelial lining as occurs with hyperlipidemia, hypertension or cigarette smoke [1-3]. Many risk factors are known for this disease and lots of biochemical markers are used to predict and so prevent it in susceptible people. Recent studies have shown that inflammation also plays a key role in atherosclerosis. C-reactive protein (CRP) has been recommended as the marker of choice to monitor cardiovascular risk, being a stronger predictor of atherosclerosis than even plasma low density lipoprotein (LDL) concentration [4,5]. Inflammation is a prominent feature of atherosclerosis [6,7], and it is postulated that as an acute-phase protein, elevation of plasma CRP may signal the underlying atherosclerotic process.

There is a relationship between some factors of coagulation system such as (fibrinogen, factor VII) and fibrinolytic factors [tissue-type plasminogen activator (t-PA) and plasminogen activator inhibitor-1 (PAI-1)] and the clinical manifestations of atherosclerosis. The relationship between fibrinogen and risk for cardiovascular events is highly proved rather than the factor VII plasma concentration. It has been proved that high levels of factor VII activity effects on the patients with high risk for coronary artery disease and myocardial infarction survivors. It seems that reduced fibrinolytic activity improve the risk of cardiovascular disease [8].

Endothelial dysfunction has been also proved to be a key variable in the pathogenesis of atherosclerosis and its complications. Nitric oxide (NO) which is produced from transformation of arginin to cytroline by iNOS and eNOS enzymes (isoformes of nitric oxide synthetase) is the most important and well known endothelium derived mediator that is responsible for endothelial dysfunction [9]. NO release by the endothelium regulates blood flow, inflammation and platelet aggregation and consequently its disruption during endothelial dysfunction can decrease plaque stability and encourage the formation of atherosclerotic lesions and thrombi [10]. In states of inflammation, NO production by the vasculature increases considerably and contributes to oxidative stress [11-13].

Flavonoids and phenolic compounds widely distributed in plants which have been reported to exert multiple biological effect, including antioxidant, free radical scavenging abilities, anti-inflammatory, anticarcinogenic, etc. Recently there has been an upsurge of interest in the therapeutic potentials of medicinal plants as antioxidants in reducing such free radical induced tissue injury [14]. Apple is one of the main sources of dietary flavonoids that show the strongest associations with decreased mortality. Also, apple is a good source of antioxidants. It contains appreciable amounts of vitamin C and of various phenolic compounds (catechins, phenolics acids, quercetin and phloretin), which also have protective effects. Epidemiological studies have linked the consumption of apples with reduced risk of some cancers, cardiovascular disease, asthma, and diabetes, lipid oxidation, cholesterol and atherosclerosis perogression. In addition quercetin has demonstrated significant anti-inflammatory activity [15]. This study was performed to determine the effects of apple juice on markers of inflammation, coagulation, endothelial and progression of atherosclerosis.

## Methods

### Preparation of the plant

First the genus and species were verified by a botanist ("*Malus Orientalis" *with herbarium number of 15811) from the Research Center of Isfahan Province Natural Resources. Then the apples were collected in Aminabad region of Isfahan. In order to standardize the apple juice, some factors such as density, vitamin C, anthocyanin and flavonoids were measured.

### Animals and experimental design

Thirty two male New Zealand rabbits with an average body weight of 2000 ± 129 g were procured from Razi Institute of Iran. The animals were acclimatized under room temperature and humidity with regular light/dark cycle for two weeks and had free access to water and a standard powdered purified diet (PARS Co, Iran) which consisted of 15% protein, 40-50% carbohydrates, 2% vegetable fat and 15-25% fiber. At the end of this period, rabbits were randomly divided into four groups of eight. Animals were fasted for 12-15 hours and venous blood samples were taken to determine baseline values. After this, each group received one of the four experimental diets: normal diet, high cholesterol diet (%1cholesterol), %1 cholesterol with 5 ml apple juice(low dose), %1 cholesterol with 10 ml apple juice(high dose) every other day. Each animal in each group had daily access to 100 g of pellets. Cholesterol (1 g for each animal, Merck)was dissolved in 2 ml olive oil given to high cholesterol diet animals by oral gavage once daily for 60 days. The same volume of olive oil was given to control animals. Then, the apple juice (5 or 10 ml)was also given orally to animals by oral gavage once daily [1,16]. The experiment lasted 60 days and the animals had unlimited access to food and water. The study was reviewed and approved by the ethics committee of Isfahan University of medical sciences. After 60 days of experiment, the blood samples were taken again.

### Measurement biochemical factors in rabbit

Blood samples were centrifuged at 3500 rpm for 20 minutes to obtain serum and plasma. The plasma was used for fibrinogen and factor VII measurement and the serum for other biomarkers.

CRP was measured using enzyme-linked immunosorbent assay kit according to manufacturer's instructions (Kamiya biomedical Co, USA) and fibrinogen was measured using coagulation kit (Mahsayaran Co, Iran). The serum level of nitrite and nitrate were measured using a colorimetric assay kit (R&D Systems, USA) that involves the Griess reaction and factor VII was measured using clotting time, in the presence of the STA-Neoplastine reagent of a system in which all the factors are present, constant and in excess except factor VII which is drived from the sample being tested (Diagnostic Stago, French).

### Assessment of the severity of atherosclerotic lesions

At the conclusion of the study, blood samples were taken from the rabbits again and overdose of sodium pentobarbital and ex-sanguinated were used to anesthetize the animals. Following chest incision, the animals' coronary of arteries were excised to study fatty streaks. After slicing and staining with hematoxylin, atherosclerotic thickness was assessed in hematoxylin stained sections on an arbitrary scale 1-4.

#### Trace

Minimal thickness of subintimal with little injury to right and left coronary endothelium.

#### Grade 1

Atherosclerotic thickness less than half as thick as the media with some form of endothelial dysfunction, macrophages and isolated foam cell inside the endothelium.

#### Grade 2

Atherosclerotic thickness half as thick as the media with accumulation of intracellular lipid, macrophage and smooth muscle cells.

#### Grade 3

Atherosclerotic thickness as thicken as the with an abundance of macrophages, smooth muscle cells and connective tissue.

#### Grade 4

Atherosclerotic thickness more that as thick as the media with a large extracellular intimal lipid core that appears as a large nucleus from the endothelial surface [17].

### Measurement physiochemical factors in apple juice

Density was meseared by densitometer, vitamin C assayed by spectrophotometeric method at 520 nm and determined photometrically with 2,4 dinitrophenyl hydrazine to form the red bis-hydrazone which is reduced to a coloreless form [18]. Total flavonoid content was measured by aluminum chloride colorimetric assay. The absorbance was measured against prepared reagent blank at 510 nm [14]. Total anthocyanin was assayed by spectrophotometeric method at 535 nm [19].

### Statistical analysis

Results are given as Mean ± SD. Data were analyzed statistically using One-Way-ANOVA test followed by LSD post test. Differences between the baseline values and the values 2 months) calculated and then used One-Way-ANOVA for comparing between groups. Then pairwise multiple comparisons were performed using LSD post test. In all instances, p value less than 0.05 was considered significant. For histological data, SPSS software was used to compare mean values between the groups. One-Way ANOVA and Tukey tests were used for histological data.

## Results

### Determination of some physiochemical factors in apple juice

After analyzing apple juice factors the amount of vitamin C was 10.7 ± 0.06 (mg/dl), total flavonoids in 100 ml of apple juice 1.36 ± 0.03 (g/100 ml equivalent catechin) and total anthocyanin in 100 g of apple juice 3.05 ± 0.85 (mg/100 g). The density was 1.037 ± 0.09 (g/cm3).

### Determination of some physiochemical factors in rabbit

Alternation in CRP, nitrite, nitrate, fibrinogen, factor VII, TC, TG, HDL-C and LDL-C concentrations in all four groups were shown in table 1. In high-cholesterol group, CRP, VII, fibrinogen, nitrite, nitrate, TC, TG and LDL-C concentrations were increased significantly compared to normal-diet group and HDL-C was decreased (p < 0.05). Both doses of apple juice significantly were decreased TC, TG, CRP, fibrinogen, factor VII levels, increased nitrite and nitrate. Also using 10 ml apple juice caused significant reduce in LDL-C and increase HDL-C in comparison with hypercholesterolemic diet, but 5 ml apple juice did not change these factors. Significant differences were observed between 5 and 10 ml apple juice groups by LDL-C(p < 0.05). No significant difference was found between 5 and 10 ml apple juice groups with regard to CRP, nitrite, nitrate, fibrinogen, factor VII, TG, HDL-C and TC concentrations. (Table 1).

**Table 1:** Comparison of LDL-C, HDL-C, TG, TC, CRP, VII, nitrite, nitrate and fibrinogen values before (baseline) and after (end of 2 months) experimental diet

**Biochemical factors**		**Groups**			
		**Cholesterolemic diet**	**5 ml apple juice with %1 chol**	**10 ml apple juice with %1 chol**	**Normal diet**
LDL-C(mg/dl)	baseline	32.50 ± 12.24	42.67 ± 15.72	52.83 ± 16.73	49.25 ± 22.47
	endof2 months	711.50 ± 102.97	498.83 ± 262.70#	214.67 ± 179.81*	50.75 ± 31.64*
HDL-C(mg/dl)	baseline	16.67 ± 3.01	23.67 ± 23.21	25.67 ± 6.38	25.25 ± 4.50
	endof2 months	58.50 ± 18.05	98.17 ± 26.91	108.83 ± 9.52*	99.33 ± 4.69*
TG(mg/dl)	baseline	148.67 ± 62.94	120 ± 65.57	164.67 ± 85.97	149.50 ± 56.18
	endof2 months	410 ± 61.61	160.83 ± 46,13*	168 ± 74.71*	150.50 ± 70.72*
TC(mg/dl)	baseline	61.8 ± 12.1	75.50 ± 55.79	86.50 ± 27.90	97.8 ± 23.7
	endof2 months	1413.67 ± 80.10	341.50 ± 121.86*	321.33 ± 212.36*	116.75 ± 19.14*
CRP(μg/ml)	baseline	2.53 ± 0.35	2.37 ± 0.28	2.33 ± 0.28	2.45 ± 0.12
	endof2 months	3.9 ± 0.21	3.29 ± 0.14*	3.20 ± 0.25*	2.63 ± 0.21*
VII(% activity)	baseline	150.17 ± 29.54	180.67 ± 40.37	199.67 ± 26.19	230 ± 2 2.06
	endof2 months	338.83 ± 78.62	245 ± 95.05*	214.5 ± 108.54*	231.5 ± 31.46*
nitrite(μmol/l)	baseline	21.3 ± 7.20	21.23 ± 4.34	25.04 ± 8.75	31.9 ± 16.10
	endof2 months	35.61 ± 10.64	62.73 ± 9.27*	70.69 ± 18.15*	27.17 11.01*
nitrate(μmol/l)	baseline	15.49 ± 2.92	14.8 ± 5.46	17.81 ± 4.23	8.11 ± 0.84
	endof2 months	22.54 ± 5.88	29.65 ± 3.96*	34.73 ± 4.52*	8.2 ± 1.63*
fibrinogen(mg/dl)	baseline	205.17 ± 20.81	243.33 ± 48.20	249.33 ± 67.24	240.75 ± 16.8
	endof2 months	293.67 ± 35.10	247.17 ± 32.30*	251 ± 53.32*	244.75 ± 13.96*

### Fatty streak formation

**Figure 1:**
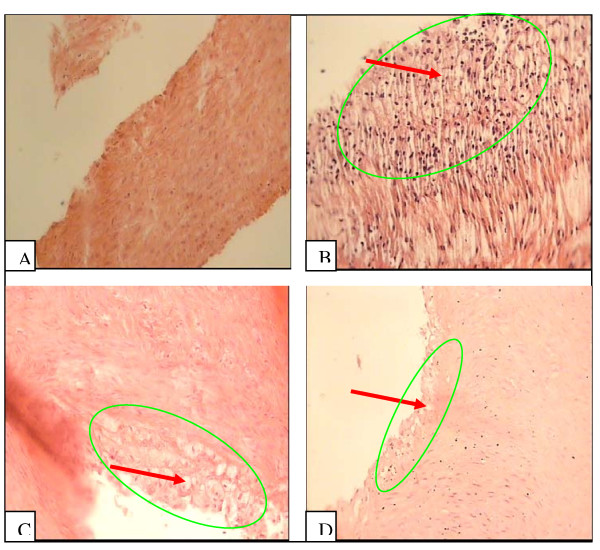
**Right Coronary artery intima cross-section in the studied groups**. Grade 1: Plaque less than half as thick as the media with some form of endothelial dysfunction. Grade 2: Plaque at least half as thick as media with accumulation of intracellular lipid, macrophages, and smooth muscle cells. Grade 3: Plaque as thick as the media with an abundance of macrophages, smooth muscle cell, and connective tissue. Grade 4: Plaque thicker than the media with a large extracellular intimal lipid core and inflammatory cell infiltration (Chekanov, 2003).

**Figure 2:**
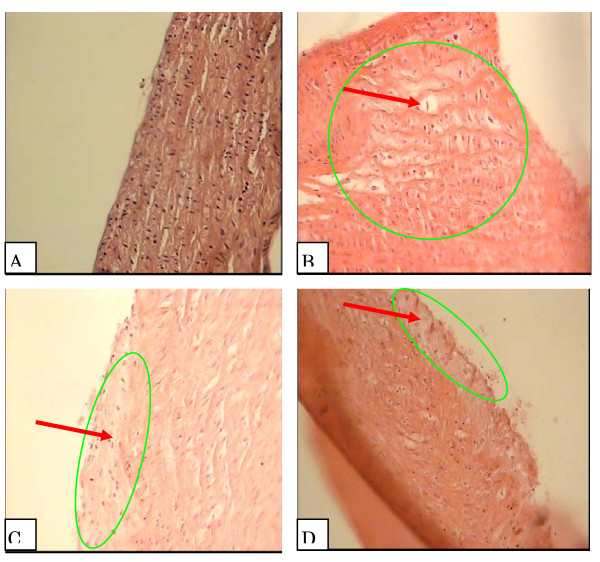
**Left Coronary artery intima cross-section in the studied groups**. Grade 1: Plaque less than half as thick as the media with some form of endothelial dysfunction. Grade 2: Plaque at least half as thick as media with accumulation of intracellular lipid, macrophages, and smooth muscle cells. Grade 3: Plaque as thick as the media with an abundance of macrophages, smooth muscle cell, and connective tissue. Grade 4: Plaque thicker than the media with a large extracellular intimal lipid core and inflammatory cell infiltration (Chekanov, 2003).

Histological sections of right and left coronary arteries stained from the 4 groups were shown in Fig 1 and 2 respectively. Atherosclerotic changes were absent in normal diet group (Fig 1a, 2a), whereas in the intimal surface of the coronary arteries from high-cholesterol diet group were seen many fat-laden macrophages. The cytoplasm of the macrophages filled with lipid droplets (foam cell) as the result of lipid digestion by the macrophage (Fig 1b, 2b). In the apple juice groups some endothelial dysfunction along a few foam cell and macrophages were seen in the intimal surface of the coronary arteries (Fig 1c, d, 2c, d). Atherosclerotic thickness grade in the apple juice groups decreased significantly compared to the high-cholesterol group (p < 0.05). In prepared incisions of from high cholesterol groups, right and left coronary arteries, the atherosclerotic thickness of right and left coronary arteries was 3.47 ± 0.37 and 3.28 ± 0.26, respectively and the plaque degree in both was 3. In 5 ml apple juice with cholesterolemic diet, the atherosclerotic thickness of right and left coronary arteries was 1.4 ± 0.26 and 1.92 ± 0.55, respectively and the plaque degree for both was 1. In 10 ml apple juice with cholesterolemic diet, the atherosclerotic thickness of right and left coronary arteries was 0.85 ± 0.36 and 0.92 ± 0.53 and plaque degree for both was 1.

## Discussion

The significant decrease in CRP, fibrinogen, factor VII, TC, TG, LDL-C and the significant increase in nitrite, nitrate and HDL-C in rabbits receiving apple juice as compared to the high-cholesterol group represent that this extract is effective in moderating the dyslipidemic condition arising from a high cholesterol diet.

Histological results indicate that apple juice significantly reduced atherosclerotic lesions of coronary arteries, when compared to the high-cholesterol groups.

In our study, nitrite and nitrate increased in high cholesterol control compared to normal diet. It has been suggested that enhanced NO synthesis might be a defense mechanism to compensate for continuous inactivation of NO and protection against damaging factors [20]. The proposed mechanism responsible for the increase of nitrite and nitrate may be a significant increase in overall NOS synthesis by other cell types (than endothelium) in advanced lesions composed of the endothelial, neuronal and inducible isoforms of nitric oxide synthase (iNOS) enzymes [21].

Both low and high-doses apple juice with cholesterolemic diet caused significant increase in nitrite and nitrate compared with high cholesterolemic diet.

The release of nitric oxide (NO) by a healthy vascular endothelium, which consists of a single layer of endothelial cells, prevents the adherence of platelets and leukocytes to the arterial wall. Therefore, reducing platelet activity could reduce athero-thrombotic events [22]. In addition to its antiatherogenic properties, NO also stimulates the vascular smooth muscle to relax and produce vasodilation. However, the endothelium can be damaged by many of the known vascular disease risk factors such as hypercholesterolemia, hypertension, and diabetes, thus reducing the bioavailability of NO[23]. The release of ROS is increased in states of oxidative stress and neutralizes the beneficial effects of NO by converting it to peroxynitrite [24]. The increase in NO and decrease in ROS attenuate multiple mechanisms that are central to the development and progression of atherosclerosis. The biological activity of NO can be effectively increased by the scavengers of oxygen-free radicals [25]. However, polyphenolic compounds can increase the NO releasing by two mechanisms: 1-Stimulation of NO synthase activity and preservation or stabilization of NO release under basal conditions. 2- Protection of NO from destruction by superoxides and other free radicals [26]. Schuldt EZ et al reported that polyphenols were able to scavenge free oxygen radicals to a limited extent resulting in an increased NO level [26].

Diets rich in dealcoholyzed red wine (DRW), quercetin or catechin induced endothelium-dependent vasorelaxation in rat aorta in a resting state through the enhancement of NO production, without modifying O2- generation, thus the bioavailability of NO was increased. The increase in the NO-cyclic GMP pathway explains the beneficial effect of flavonoids at vascular level [27].

Following concurrent use of 5 and 10 ml apple juice with cholesterolemic diet, fibrinogen and factor VII levels were significantly decreased in comparison with high-cholesterol group.

Studies show that red wine consumption caused significant decreases in fibrinogen and factor VII [28,29].

In a study by Grenett HE et al in rats, the impact of polyphenols on expression of fibrinolytic protein [urokinase-pA(u-PA), (t-PA), (PAI-1)] mRNAs in endothelial cells of aorta was evaluated. The expression of t-PA and u-PA mRNA was up-regulated, but conversely PAI-1 mRNA expression was down-regulated by both ethanol and polyphenols including catechin and quercetin. As a result, the firbrinolysis was increased in rat endothelial cells, an effect which might exert a beneficial cardio protective role [30].

The individual polyphenols of apple juice and ciders were examined for their ability to inhibit tissue activator, urokinase and plasmin. Neither phloridzin nor chlorogenic acid had any inhibitory activity at concentrations of 500 micrograms/ml while epicatechin had only a slight inhibitory effect at this concentration [31].

Using both doses of apple juice with cholesterolemic diet induced a significant decrease in CRP compared to hypercholesterolemic diet. In high cholesterol group, CRP was increased significantly compared to the normal diet group; however it has been shown in another studies with hypercholesterolemic rabbits [32,33].

C-RP may actively promote atherogenesis [34], causing lesion formation via mechanisms such as endothelial dysfunction and leukocyte activation [35] and changes in plaque structure resulting in stability reduction and enhancing rupture. Many reviews aimed to study the effects of flavonoids on inflammatory processes [36] which showed that they may inhibit some active enzymes during the inflammatory process [37]. Isoforms of iNOS and of cyclooxygenase (COX-2) are responsible for the production of prostaglandins and nitric oxide biosynthesis which act as a mediators. In vitro studies have demonstrated that nitric oxide production and the expression of iNOS inhibit by flavonoid quercetin [38]. Flavonoids can modulate the molecular events cascade in several critical steps resulted in iNOS or COX-2 over expression. It seems that activation of a transcription essential for the expression of proinflammatory genes, the nuclear factor kappa B (NF-kappa B)converge the iNOS and COX-2 induction pathways [39].

Some of inducible transcription factors can constitute potential key targets for the treatment of the inflammation and NFkappaB is one of the most important of them which can trigger a cascade of molecular events by its modulation. It is showed that CRP protein level in hepatic cells may reduce by diverse flavonoides and that the effect is dose-dependent [40]. Studies indicated that CRP can be induced by IL-6 mechanism that involves NF-kappaB activation [41,42]. An inhibition of the nuclear NF-kappaB translocation and of CRP synthesis in hepatocytes can cause by alterations in the intracellular state redox [43]. So it is possible that effects of flavonoids on CRP expression could be mediated, at least partly, by the modulation of the NF-kappaB-dependent pathway.

Results of Golzarand M et al showed that ingestion of apple cider vinegar in patients with type 2 diabetes had no effect on CRP but it reduced IL-6 concentrations [44].

Findings of Chung OK, et al demonstrated that intake of dietary flavonoids is inversely associated with serum CRP concentrations in U.S. adults. Intake of flavonoid-rich foods may reduce inflammation-mediated chronic diseases [45].

Studies of Terra X, et al indicated that the procyanidins of grape seed decrease plasma CRP levels in rat. The decrease in plasma CRP in hypercholesterolemic rats is related to a down-regulation of CRP mRNA expression in the liver [46].

We showed that dietary consumption of 5 and 10 ml apple juice with cholesterol diet significantly decreased serum TC and TG. Aprikian O, et al [2001] found that when cholesterol fed rats were supplemented with lyophilized apples, there was a significant drop in plasma cholesterol and liver cholesterols and an increase in high-density lipoproteins (HDL). Furthermore, they found that cholesterol excretion increased in the feces of rats fed apples, suggesting reduced cholesterol absorption [47]. Also, Aprikian O, et al [2003] in more recent studies, found that combined apple pectin and apple phenolic fractions lowered plasma and liver cholesterol, triglycerides, and apparent cholesterol absorption to a much greater extent than either apple pectin alone or apple phenolics alone [48].

Histological results indicate that apple juice intake reduced atherosclerotic lesion in coronary vessels significantly with compared to hypercholesterolemic group. Because inflammation has an important role in atherosclerosis development, significant reduction in inflammatory lesion may be due to anti-inflammatory effect of apple juice.

The protective effects of flavonoids against chronic diseases have been attributed to their free radical-scavenging property. In the case of CVD, flavonoids have been shown to reduce low density lipoprotein (LDL) oxidation, an important step in atherogenesis [49,50]. Hung MT, et al [51] has suggested that polyphenolic compounds inhibit proinflammatory cytokines expression including IL-6, IL-1 and CRP expression. Apple was identified as a major dietary source of flavonoids especially flavan-3-ols (catechins) in the epidemiologic studies that inhibits cyclooxygenase enzymes [52,53] that produce inflammation and stimulate inflammatory responses in body [54].

Studies by Decorde K, et al indicated that phenols of grape, apple and their juices reduce atherosclerosis lesion in hamster. This value was reduced by %93 in grape juice,%78 in purple grape,%60 in apple juice and %48 in apple[16].

In study performed by Da Luz PL, et al on the effect of red wine on experimental atherosclerosis, the rabbits that given red wine had significantly less atherosclerotic plaque development in their arteries than those not given wine at all[55].

In conclusion, results of this study suggest that hypercholesterolemic atherosclerosis is associated with an increase in oxidative stress in coronary arteries and that apple juice is effective in reducing hypercholesterolemic atherosclerosis by lowering levels of fibrinogen, VII, CRP, TC, TG, LDL-C and raising levels of nitrite, nitrate and HDL-C. Therefore, apple juice may be useful in preventing hypercholesterolemic atherosclerosis and lowering the related risk of coronary artery disease.

## Competing interests

The authors declare that they have no competing interests.

## Authors' contributions

AHR participated in the sequence alignment and drafted the manuscript, NE carried out the laboratory tests. AE participated in the sequence alignment. MS participated in the design of the study and performed the statistical analysis. SA conceived the study, and participated in its design and coordination. All authors read and approved the final manuscript.

## References

[B1] Asgary S, Jafari Dinani N, Madani H, Mahzoni P, Naderi GH (2006). Effect of *Glycyrrhiza glabra *extract on aorta wall atherosclerotic lesion in hypercholesterolemic rabbits. Pak J Nutr.

[B2] Julie H, Campbell M, Johnny L, Efendy J, Gordon R (2001). Molecular basis by which garlic suppresses atherosclerosis. J Nutr.

[B3] Crowther MA (2005). Pathogenesis of atherosclerosis. The American Society of Hematology.

[B4] Libby P (2002). Inflammation in atherosclerosis. Nature.

[B5] Pearson TA, Mensah GA, Alexander RW (2003). Markers of inflammation and cardiovascular disease: application to clinical and public health practice: a statement for healthcare professionals from the Centers for Disease Control and Prevention and the American Heart Association. Circulation.

[B6] Ridker PM, Rifai N, Rose L (2002). Comparison of C-reactive protein and low-density lipoprotein cholesterol levels in the prediction of first cardiovascular events. N Engl J Med.

[B7] Ross R (1999). Atherosclerosis: an inflammatory disease. N Engl J Med.

[B8] Gensini GF, Comeglio M, Colcila A (1996). Hemostatic factors, atherogenesis and atherosclerosis. Biomedecine & Pharmacotherap.

[B9] Moncada S, Martin JF (1993). Evolution of nitric oxide. Lancet.

[B10] Vane JR, Anggard EE, Botting RM (1990). Regulatory functions of the vascular endothelium. N Engl J Med.

[B11] Heitzer T, Schlinzig T, Krohn K, Meinertz T, Munzel T (2001). Endothelial dysfunction, oxidative stress, and risk of cardiovascular events in patients with coronary artery disease. Circulation.

[B12] Schachinger V, Britten MB, Zeiher AM (2000). Prognostic impact of coronary vasodilator dysfunction on adverse long-term outcome of coronary heart disease. Circulation.

[B13] Cai H, Harrison DG (2000). Endothelial dysfunction in cardiovascular diseases: the role of oxidant stress. Circ Res.

[B14] Kumar S, Kumar D, Rakash O (2008). Evaluation of antioxidant potential, phenolic and flavonoid contents of hibiscus tiliaceus flowers. EJAFche.

[B15] Boyer J, Liu RH (2004). Apple phytochemicals and their health benefits. Nutr J.

[B16] Decorde K, Teissedre PL, Auqer C, Cristol JP, Rouanet JM (2008). Phenolics from purple grape, apple, purple grape juice, and apple juice prevent early atherosclerosis induced by an atherogenic diet in hamsters. Mol Nutr Food Res.

[B17] Chekanov VS (2003). Low frequency electrical impulses reduce atherosclerosis in cholesterol fed rabbits. Med Sci Monit.

[B18] Mccormick DB, Greene HL, Carl A, Britis Edward R, Ashwood (1994). Vitamins. Tietz Text Book of Clinical Chemistry.

[B19] Francis FJ (1982). In anthocyanins as food colors.

[B20] Ferlito S, Gallina M, Catassi S, Bisicchia A, Di Salvo MM (1999). Nitrite plasma levels in normolipemic and hypercholesterolemic patients with peripheral occlusive arteriopathy. Panminerva Med.

[B21] Tang FT, Qian ZY, Liu PQ, Zheng SG, He SY, Bao LP, Huang HQ (2006). Crocetin improves endothelium-dependent relaxation of thoracic aorta in hypercholesterolemic rabbit by increasing eNOS activity. Biochem Pharmacol.

[B22] Viana M, Barbas C, Bonet B, Bonet MV, Castro M, Fraile MV, Herrera E (1996). In vitro effects of a flavonoid-rich extract on LDL oxidation. Atherosclerosis.

[B23] Vita JA, Keaney JF (2002). Endothelial function: a barometer for cardiovascular risk?. Circulation.

[B24] Aldini G, Carini M, Piccoli A, Rossoni G, Facino RM (2003). Procyanidins from grape seeds protect endothelial cells from peroxynitrite damage and enhance endothelium-dependent relaxation in human artery: new evidences for cardio-protection. Life Sci.

[B25] Bouloumie A, Bauersachs J, Linz W, Schölkens BA, Wiemer G, Fleming I, Busse R (1997). Endothelial dysfunction coincides with an enhanced nitric oxide synthase expression and superoxide anion production. Hypertension.

[B26] Schuldt EZ, Ckless K, Simas ME, Farias MR, Riberio-Do-Valle RM (2000). Butanolic fraction from Cuphea carthagenensis Jacq McBride relaxes rat thoracic aorta through endothelium- dependent and endothelium independent. J Cardiovasc Pharmacol.

[B27] Benito S, Lopez D, Sáiz MP, Buxaderas S, Sánchez J, Puig-Parellada P, Mitjavila M (2002). A flavonoid-rich diet increases nitric oxide production in rat aorta. Br J Pharmacol.

[B28] Mukamal KJ, Jadhav PP, DAgostino RB, Massaro JM, Mittleman MA, Lipinska I, Sutherland PA, Mathney T, Levy D, Wilson PW, Ellison RC, Silbershatz H, Muller JE (2001). Alcohol consumption and haemostatic factors: analysis of the Framingham offspring cohort. Circulation.

[B29] Yarnell JW, Sweetnam PM, Rumley A, Lowe GD (2000). Lifestyle and hemostatic risk factors for ischemic heart disease: the caerphilly study. Arterioscler Thromb Vasc Biol.

[B30] Grenett HE, Abou-Agag LA, Parks DA (2004). Ethanol and polyphenols (CAT, QUER) increase expression of fibrinolytic protein mRNAs in vivo in rat aortic endothelium. Biol Re.

[B31] Ogston D, Lea AG, Langhorne P, Wilson SB (1985). The influence of the polyphenols of cider on plasmin and plasminogen activators. Br J Haematol.

[B32] Jialal I, Devaraj S, Singh U (2006). Sources of CRP in atherosclerotic lesions. Am J Pathol.

[B33] Haghjooyjavanmard SH, Nematbakhsh M, Monajemi A, Soleimani M (2008). Von willebrand factor, Creactive protein, nitric oxide and vascular endothelial growth factor in a dietary reversal model of hypercholesterolemia in rabbit. Biomed Papmed fac univ palacky olomus Czech Rebub.

[B34] Torzewski M, Rist C (2000). Mortensen RFC-reactive protein in the arterial intima: role of C-reactive protein receptor-dependent monocyte recruitment in atherogenesis. Arterioscler Thromb Vasc Biol.

[B35] Pasceri V, Willerson JT, Yeh ETH (2000). Direct proinflammatory effect of C-reactive protein on human endothelial cells. Circulation.

[B36] Nair MP, Mahajan S, Reynolds JL, Aalinkeel R, Nair H, Schwartz SA, Kandaswami C (2006). The flavonoid quercetin inhibits proinflammatory cytokine (tumor necrosis factor alpha) gene expression in normal peripheral mononuclear cells via modulation of the NF-kappa beta system. Clin Vaccine Immunol.

[B37] Kwon KH, Murakami A, Tanaka T, Ohigashi H (2005). Dietary rutin, but not its aglycon quercetin, ameliorates dextran sulfate sodium-induced experimental colitis in mice: attenuation of proinflammatory gene expression. Biochem Pharmacol.

[B38] Martinez-Flores S, Gutierrez-Fernandez B, Sanchez-Camps S, Gonzalez-Gallego J (2005). Quercetin prevents nitric oxide production and nuclear factor kappa B activation in interlekin 1-β activated rat hepatocytes. J Nutr.

[B39] García-Mediavilla V, Crespo I, Collado PS, Esteller A, Sánchez-Campos S, Tuñón MJ, González-Gallego J (2007). The anti-inflammatory flavones quercetin and kaempferol cause inhibition of inducible nitric oxide synthase, cyclooxygenase-2 and reactive C-protein, and down-regulation of the nuclear factor kappaB pathway in Chang Liver cells. Eur J Pharmacol.

[B40] Jiang B, Xu S, Hou X, Pimentel DR, Brecher P, Cohen RA (2004). Temporal control of NF-kappaB activation by ERK differentially regulates interleukin-1beta-induced gene expression. J Biol Chem.

[B41] Ahmad N, Chen LC, Gordon MA, Laskin JD, Laskin DL (2002). Regulation of cyclooxygenase-2 by nitric oxide in activated hepatic macrophages during acute endotoxemia. J Leuk Biol.

[B42] Odontuya G, Hoult JRS, Houghton PJ (2005). Structure-activity relationship for anti-inflammatory effect of luteolin and its derived glycosides. Phytother Res.

[B43] Maehira F, Miyagi I, Eguchi Y (2003). Selenium regulates transcription factor NF-kappaB activation during the acute phase reaction. Clin Chim Acta.

[B44] Golzarand M, Ebrahimi-Mamaghani M, Arefhosseini SR (2008). Effect of processed Berberis vulgaris in apple vinegar on blood pressure and inflammatory markers in type 2 diabetic patients. Iranian Journal of Diabetes and Lipid Disorders.

[B45] Chung OK, Chang SJ, Claycombe KJ, Song WO (2008). Serum c-reactive protein concentrations are inversely associated with dietary flavonoid intake in U.S. adults. J Nutr.

[B46] Terra X, Montagut G, Bustos M, Liopiz N, Ardevol A, Blade C, Fernandez-Larrea J, Pujadas G (2009). Grape seed procyanidins prevent low grade inflammatory by modulating cytokine expression in rats fed a high fat diet. J Nutr Biochem.

[B47] Aprikian O, Levrat-Verny M, Besson C, Busserolles J, Remesy C, Demigne C (2001). Apple favourably affects parameters of cholesterol metabolism and of anti-oxidative protection in cholesterol fed rats. Food Chem.

[B48] Aprikian O, Duclos V, Guyot S, Besson C, Manach C, Bernalier A, Morand C, Remesy C, Demigne C (2003). Apple pectin and a polyphenol rich apple concentrate are more effective together than separately on cecal fermentations and plasma lipids inrats. J Nutr.

[B49] De Whalley CV, Rankin SM, Hoult JR, Jessup W, Leake DS (1990). Flavonoids inhibit the antioxidative modification of low density lipoproteins. Biochem Pharmacol.

[B50] Hayek T, Fuhrman B, Vaya J, Rosenblat M, Belinky P, Coleman R, Elis A, Aviram M (1997). Reduced progression of atherosclerosis in apoprotein E-deficient mice following consumption of red wine, or its polyphenols quercetin or catechin, is associated with reduced susceptibility of LDL to oxidation and aggregation. Arterioscler Thromb Vasc Biol.

[B51] Huang MT, Liu Y, Ramji D, Lo CY, Ghai G, Dushenkov S (2006). Inhibitory effects of Black Tea Theaflavin Derivatives on 12-O-tetradecanoylphorbo l-13-acetate-induced inflammation and arachidonic acid metabolism in mouse ears. Mol Nutr Food Res.

[B52] Prior RL, Shils ME, Shike M, Ross AC, Caballero B, Cousins R (2006). Phytochemicals. Modern Nutrition in Health and Disease.

[B53] Lee JY, Jang YW, Kang HS, Moon H, Sim SS, Kim CJ (2006). Anti-inflammatory action of phenolic compounds from Gastrodia Elata Root. Arch Pharm Res.

[B54] Duncan K (2007). Musculoskeletal and Collagen Disorders. Escott-Stump S Nutrition and Diagnosis Related Care.

[B55] Da Luz PL, Serrano Jr C, Chacra AP, Monterio HP, Yoshida VM, Futado M, Ferreria S, Gutierrez P, Pileggi F (1999). The effect of red wine on experimental atherosclerosis: lipid-independent protection. Exp Mol Pathol.

